# Genetic polymorphism at an odorant receptor gene (Or39) among mosquitoes of the *Anopheles gambiae* complex in Senegal (West Africa)

**DOI:** 10.1186/1756-0500-7-321

**Published:** 2014-05-30

**Authors:** Audrey Arnal, Pierre Kengne, Cecile Brengues, Kounbobr Roch Dabire, Abdoulaye Diabate, Hubert Bassene, Frederic Simard

**Affiliations:** 1 Institut de Recherche pour le Développement (IRD), UMR IRD 224-CNRS 5290-UM1-UM2 MIVEGEC (Maladies Infectieuses et Vecteurs: Ecologie, Genetique, Evolution et Contrôle), team BEES (Biology, Ecology and Evolution of vector Systems), 911 Avenue Agropolis, BP 64501, Montpellier, cedex 5 34394, France; 2 Organisation de Coordination pour la lutte contre les Endémies en Afrique Centrale (OCEAC), P.O. Box 288, Yaoundé, Cameroon; 3 Institut de Recherche en Sciences de la Santé (IRSS), P.O. Box 545, Bobo-Dioulasso, Burkina Faso; 4 UMR 198 URMITE, Campus International IRD/UCAD de Hann, Dakar, Senegal

**Keywords:** Mosquito, Malaria, *Anopheles gambiae*, Speciation, Olfactory receptor

## Abstract

**Background:**

Olfaction plays a significant role in insect behavior during critical steps of their life-cycle, such as host-seeking during foraging or the search for a mate. Here, we explored genetic polymorphism within and divergence between sibling species of the African malaria mosquito, *Anopheles gambiae sensu lato* in the gene sequence and encoded peptides of an odorant receptor, Or39. This study included sympatric specimens of *An. gambiae sensu stricto, An. coluzzii* and *An. arabiensis* sampled together in the village of Dielmo, Senegal.

**Results:**

A 1,601 bp genomic sequence composed of 6 exons and 5 introns was obtained for Or39 from 6–8 mosquitoes in each of the 3 species. DNA sequence analysis revealed a high level of molecular polymorphism (π = 0.0154; Haplotype diversity = 0.867) and high overall genetic differentiation between taxa (Fst > 0.92, P < 0.01). In total, 50 parsimony informative sites were recorded. Throughout the whole dataset, there were 13 non-synonymous mutations resulting in aminoacid changes in the encoded protein. Each of the 6 different identified peptides was species-specific and none was shared across species. Most aminoacid changes were located on the intracellular domains of the protein. However, intraspecific polymorphisms in *An. gambiae* and *An. arabiensis* as well as species-specific mutations also occurred in the first extracellular domain.

**Conclusions:**

Although obtained from a limited number of specimens, our results point towards genetic differences between cryptic species within the *An. gambiae* complex in a gene of biological relevance that might be of evolutionary significance when exposed to disruptive selective forces.

## Background

The *Anopheles gambiae sensu lato* complex groups together 8 sibling species, including 2 of the most powerful African human malaria vectors, *An. arabiensis* (Patton, 1905) and *An. gambiae sensu stricto* (Gile, 1902). The latter comprises 2 incipient species named *An. gambiae* (formerly, *An. gambiae* S form) and *An. coluzzii* (formerly, *An. gambiae* M form) which are genetically and biologically isolated from one another through assortative mating [1-6]. Population genetics and genomics studies revealed little genetic divergence throughout the genome, except in a few discrete regions with low recombination, a pattern that is compatible with retention of ancestral polymorphism after recent speciation and/or non negligible levels of residual gene flow between incipient species [3,7-11]. Indeed, although hybrids are rarely observed in the wild, no intrinsic fitness reduction was found when hybrids are artificially produced in the laboratory [12]. Thus, prezygotic barriers are believed to play a major role in fostering divergence between *An. coluzzii* and *An. gambiae* through strong assortative mating. These mosquitoes mate in swarms and further studies using the previous molecular forms’ nomenclature have shown that swarming and mating mainly involved mosquitoes of the same form [13-15] and that males and females engage in close-range acoustic interactions by shifting their flight tones to match each other prior to copulation [16]. However, the cues perceived by virgin mosquitoes for long distance orientation towards mating areas in search for a mate are still largely unknown. Close-range interactions between potential mating pairs are still incompletely understood as well. These processes may be mediated by volatile compounds like pheromones, which were shown to be involved in the mating behavior of several biting flies [17,18]. The olfactory system, including odorant receptors, would therefore play a critical role in detecting biologically active compounds such as volatiles emanating from potential mates, other insects (conspecifics, predators), hosts or candidate oviposition sites [19-21]. Odorant receptor (OR) genes have been identified in genomic regions of high differentiation between *An. gambiae* and *An. coluzzii*[3,22-24]. One of these OR genes, AgOr39 [AGAP 002639 in VectorBase, thereafter Or39] located on chromosome 2R, is highly polymorphic. Recent analyses of a c.a. 400 bp partial sequence of the gene suggested directional selection acting on this gene in Cameroon’s *An. coluzzii*, where reproductive isolation between *An. gambiae* and *An. coluzzii* is highest [8,9,24]. This pattern was not observed in specimens collected from Mali [24]. Therefore, the authors suggested that this locus might be related to specific processes of ecological divergence prompting assortative mating among *An. gambiae* and *An. coluzzii* sympatric populations in only a limited area within their overlapping distribution range [24]. Different populations of the 2 incipient species are indeed known to exhibit different levels of reproductive isolation in different geographic locations across their range [10,11,25].

Here, we provide new data on Or39 molecular polymorphism by i) expanding the breadth of the sequencing effort to cover a larger portion of the gene, including 6 exons and 5 introns, and ii) exploring molecular polymorphism and divergence in this gene among specimens of *An. gambiae*, *An. coluzzii* and *An. arabiensis* collected in Senegal (West Africa), a geographic area where the 3 species co-exist and where genetic admixture between *An. gambiae* and *An. coluzzii* has been reported [11,25,26].

## Methods

### Study site and mosquitoes

Fieldwork was carried out in agreement with the procedures of the National Ethics Committee in Senegal (clearance N^o^ 1971 MPM/DS/DER). Our study complies with the Convention on Biological Diversity (http://www.cbd.int/convention/) and the Convention on International Trade in Endangered Species of wild fauna and flora threatened with extinction (http://www.cites.org/).

Mosquitoes used for the study were collected by landing catches on adult volunteers between July and October 2007 (rainy season) in the village of Dielmo (13°45′N, 16°25′W) situated 285 km southeast of Dakar (Senegal) and about 10 km north of the Gambian border. Dielmo is a village of about 350 inhabitants bordered by a semi-permanent freshwater river. In Dielmo, *An. arabiensis*, *An. coluzzii* and *An. gambiae s.s.* occur together all year round, although with seasonal fluctuation in their relative abundance and prevalence of *An. gambiae*/*An. coluzzii* hybrids reaching 3% during the rainy season [26]. After collection, mosquitoes were identified in the field as *An. gambiae s.l.* using morphological keys [27,28]. Only females of *An. gambiae s.l.* were included in subsequent analyses.

### DNA extraction and molecular identification

Genomic DNA was extracted from legs or whole mosquito bodies using Cetyl Trimethyl Ammonium Bromide (CTAB) following the protocol of Morlais *et al.*[29]. DNA pellets were dried out and re-suspended in 20 μl of nuclease-free water and stored at -20°C. Mosquito species within the *An. gambiae s.l.* complex were identified using a standard PCR-RFLP protocol for molecular identification of *An. gambiae s.s., An. coluzzii, An. arabiensis* as well as F1 hybrid specimens [30].

### PCR amplification and sequencing of Or39

PCR reactions were carried out using 10–20 ng of template DNA in 25 μl reaction containing 0.5 Unit Taq polymerase (Quiagen, Courteboeuf, France) in manufacturer’s buffer, 1.5 mM MgCl2, 200 μM each dNTP (PE Applied Biosystems) and 10 pmol each forward and reverse primers.

Primers were designed from the *An. gambiae s.s.* gene annotation AGAP002639 in VectorBase (https://www.vectorbase.org/) using Primer3 software [31]. A 1.6 kb region encompassing the whole transcribed region of the Or39 gene was amplified using forward primer Or39F (5′-GGTGCTGCAGCTTCTAATC-3′) and reverse primer Or39R (5′-CAAAAAGGACTTCATCAGTG-3′). Cycling conditions for amplification included denaturation at 94°C for 5 min, followed by 35 cycles at 94°C for 30 s, 50°C for 30 s and 72°C for 1 min, with a final extension step at 72°C for 7 min. PCR products were examined on a 1.5% agarose gel, and cloned using the Zero Blunt TOPO PCR Cloning Kit (Invitrogen, Paisley, UK). Individual transformed colonies were selected and fragments of the appropriate size were sequenced using PE BigDye Terminator Ready Reaction Kit (PE Applied Biosystems) on an ABI 3130XL apparatus (Applied Biosystems, France) according to manufacturer’s instructions. To avoid sampling bias, a single allele (haplotype sequence) was arbitrarily selected from each specimen for analysis. Furthermore, 2 to 3 clones of the expected size were randomly sequenced per specimen, in order to check for PCR accuracy prior to cloning through double sequencing of the same allele (see [32] for more details).

### Sequence analysis

Sequences were visually checked and aligned using Seqscape software (Applied Biosystems, France). Aligned DNA sequences were imported into MEGA6 [33] to compute basic sequence statistics, assess genetic distance between groups and build a Neighbour-Joining tree. To determine whether signatures of natural selection were present in our dataset, we analyzed the relative rates of non-synonymous and synonymous substitution (dN/dS) and performed the MacDonald-Krietman test of neutral evolution as implemented in MEGA6. Genetic differentiation between mosquito populations was assessed by Fst estimates and tested by permutation tests in MEGA6. The hydrophobic regions on the encoded receptor protein were identified using Kyte-Doolittle hydropathy algorithm [34] as implemented through the website of the Pasteur Institute (http://mobyle.pasteur.fr, TopPred 1.10, [35]).

## Results

### Species diversity and sequence analysis

Field collections returned 224 females of *Anopheles s.l.* among which 42% (N = 94) were *An. arabiensis*, 39% (N = 87) were *An. gambiae*, 11% (N = 24) were *An. coluzzii* and, 1% (N = 2) were *An. gambiae*/*An. coluzzii* F1 hybrids. The remaining 7% (N = 17) failed to amplify and were not considered in the study. All 3 species were found together in the collections from July to September, during the peak of the malaria transmission period. The 2 hybrid specimens were collected in July, and no *An. coluzzii* specimen was collected in October and November.

A 1,608-bp DNA sequence was obtained for the entire Or39 gene from 7 *An. gambiae* and 6 *An. coluzzii* specimens. The sequences matched the reference genomic sequence published from the PEST (Pink Eye STandard) strain of *An. gambiae* s.s. [AGAP002639] in VectorBase. The exon/intron structure of the gene was determined by reference to the cDNA sequence available in VectorBase (Transcript ID: AGAP0002639-RA). Full-length Or39 sequences consisted of 6 exons separated by 5 non-coding introns. Sequences obtained from 8 *An. arabiensis* specimens were shorter, due to a 7-bp deletion shared by all specimens, located in a non-coding region of the gene (Intron 1, between positions 430–437). Only partial sequence fragments were retrieved from the two hybrid specimens, which were not included in further analyses.

### Genetic polymorphism and divergence

**Figure 1:**
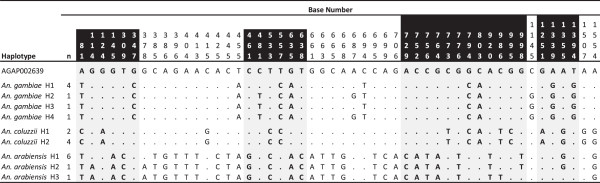
**Summary sequence alignment of the different Or39 haplotypes detected in mosquito specimens collected in the locality of Dielmo in Senegal during the 2007 rainy season.** Only parsimony informative sites are shown (N = 50). Nucleotides are numbered according to the published reference sequence from the PEST strain of *An. gambiae* (VectorBase, AGAP002639). Dots indicate identity to the reference sequence. The coding regions (exons) are shaded.

DNA polymorphism analysis identified 68 (4.25%) variable sites along the sequence, of which 50 were parsimony informative (Figure 1). Average nucleotide diversity was lowest in *An. coluzzii* (π = 0.00033) and one order of magnitude higher in *An. gambiae* and *An. arabiensis* (π = 0.0012 and 0.0025, respectively) (Table 1). However, haplotype diversity was lower in *An. arabiensis* (Hd = 0.464) than in *An. gambiae* and *An. coluzzii* (Hd = 0.533 and 0.714, respectively). Throughout the whole gene region, there were 33 SNPs differences between *An. gambiae* and *An. arabiensis,* and 35 between *An. arabiensis* and *An. coluzzii*. These mutations were located in both exon and intron domains (Figure 1). A total of 16 SNPs were observed between *An. gambiae* and *An. coluzzii*, including 11 in exons. Four of these were replacement mutations. Within the coding region (Total exon length = 1,224 bp), non-synonymous diversity (dN, π nonSyn = 0.005) was 6 times lower than synonymous diversity (dS, π Syn = 0.029), although the difference was not significant (P> > 0.05, two-sided Fisher test of neutral evolution available in MEGA6). There was no evidence for deviation from neutral expectations in any species (McDonald-Kreitman test, P > 0.39).

**Table 1:** **Summary statistics for polymorphism in the Or39 receptor gene within sympatric populations of *An. gambiae, An. coluzzii *** **and *An. arabiensis *** **from Dielmo, Senegal**

**Population**	**n**	**size (bp)**	**s**	**h**	**π**	**Hd**	**D**
*An. gambiae*	7	1601	4	4	0.0012	0.714	0.79 ns
*An. coluzzii*	6	1601	1	2	0.0003	0.533	0.85 ns
*An. arabiensis*	8	1601	13	3	0.0025	0.464	-1.06 ns
All	21	1601	61	9	0.0154	0.867	1.23 ns

n, number of DNA sequences; s, number of segregating sites; h, number of haplotypes based on the number of segregating sites; π, nucleotide diversity; Hd, haplotype diversity; D, Tajima’s (1989) statistic; ns, not significant.

At the DNA level, divergence between the 3 species was highly significant (P < 0.01) with Fst estimates above 0.92 across the whole gene.

### Phylogenetic relationships among taxa

**Figure 2:**
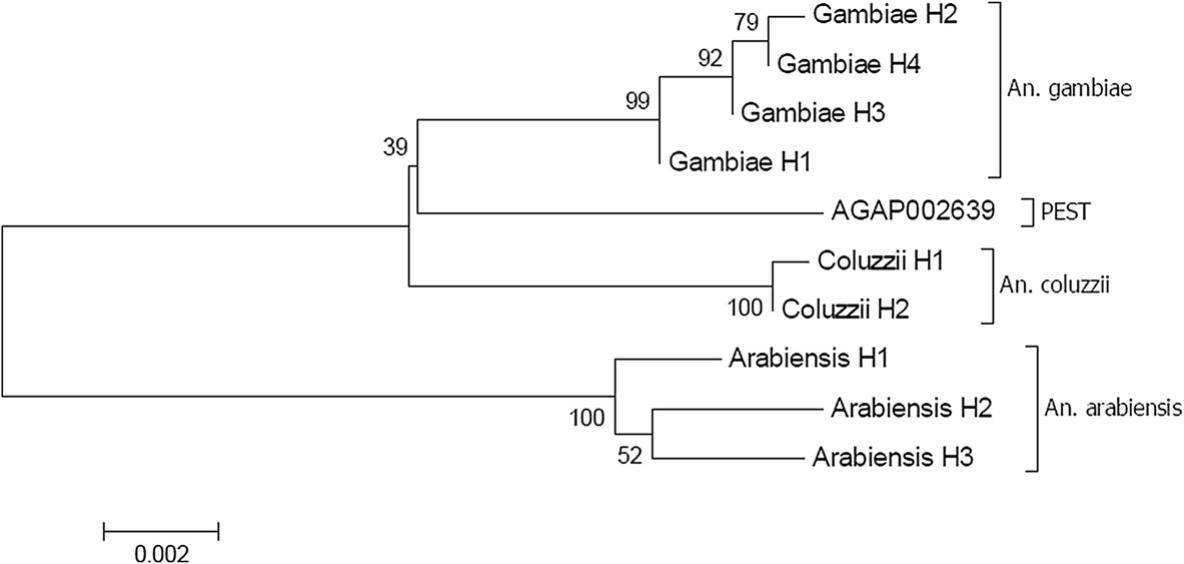
**A Neighbor-Joining tree based on molecular polymorphism in intron and exon sequences (1,601 bp) of the Or39 odorant receptor gene among mosquito specimens collected in the locality of Dielmo, Senegal.** Values at the nodes are bootstrap values [36].

Figure 2 shows a Neighbour-Joining tree constructed with Kimura-2-parameter genetic distances, retained as the best model based on the Bayesian Information Criterion (BIC) scores used in MEGA6, between the 10 haplotypes shown in Figure 1. Specimens segregated unambiguously into the 3 known taxa, *An. gambiae*, *An. coluzzii* and *An. arabiensis*. Note that the PEST strain sequence maps as expected, in between the *An. gambiae* and *An. coluzzii* clusters. The PEST genome is indeed known to be a composite genome of the two cryptic species.

### Peptide analysis

**Figure 3:**
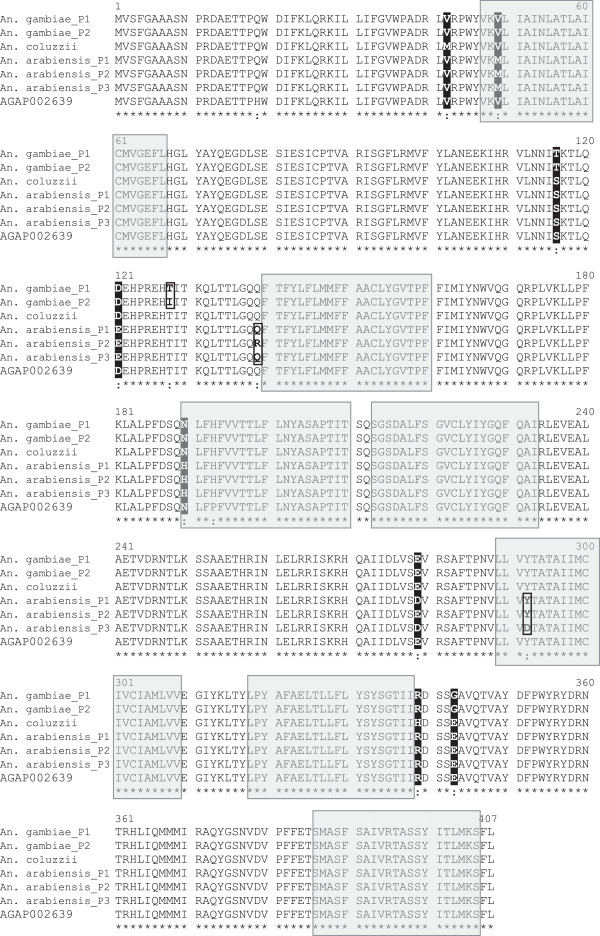
**Alignment of the predicted amino acid sequences for Or39 odorant receptor of *An. gambiae, An. coluzzii *and *An. arabiensis *from Dielmo, Senegal.** Reference amino acid sequence for the PEST strain of *An. gambiae* was obtained from the published cDNA sequence (Accession N° XM_312289.1). Shaded areas represent hydrophobic trans-membrane domains of the protein. Stars denote identity to the reference sequence and dots show amino acid changes. Within-species polymorphisms are bolded and boxed; Polymorphic sites among species are highlighted in reversed fonts.

The open reading frame of the 1400 bp cDNA encoded a 407 amino acid sequence for Or39. Throughout the whole dataset, there were 13 non-synonymous replacement mutations resulting in aminoacid changes in the encoded protein. The amino acid sequences obtained from deducted cDNA sequences resulted in 6 distinct peptides: 3 in *An. arabiensis*, 2 in *An. gambiae* and a single peptide encoded by *An. coluzzii* DNA (Figure 3). Note that all peptides identified from wild mosquito specimens were distinct from the protein sequence deducted from the PEST strain DNA. Moreover, each peptide was species-specific and they were not shared across species. There were 4 amino acid changes between *An. gambiae* and *An. coluzzii* resulting in a Methionin-to-Valine substitution at position 42, a Serine-to-Threonine substitution at position 116, a Histidine-to-Arginine substitution at position 339 and a Glutamine-to-Glycine substitution at position 343 (Figure 3).

### Structural analysis of the encoded protein

The structural analysis of the encoded protein explored through the Kyte-Doolittle hydropathy algorithm returned a single most likely topology for Or39 with 7 trans-membrane domains that were reported at the same position in the 3 species (Figure 3). Given the known membrane conformation for odorant receptors with the N-terminus internal and the C-terminus external [37], the topology with 4 intra-cellular domains and 4 extra-cellular domains is most likely. Accordingly, most aminoacids changes on the receptor were located on the intracellular domains of the protein. However, intraspecific polymorphisms in *An. gambiae* and *An. arabiensis* as well as species-specific mutations also occur in the first (long) extracellular domain (Figure 3).

## Discussion and conclusion

The study of reproductive behavior in *An. gambiae s.l.* is of major importance in the understanding of how the main vectors of malaria in Africa have evolved and how selective pressures operate to foster divergence and/or gene flow within as well as among these cryptic taxa.

In the village of Dielmo in Senegal, *An. arabiensis, An. coluzzii* and *An. gambiae s.s.* occur in sympatry throughout the year, although with seasonal fluctuation in their relative frequencies [27,38]. The prevalence of F1 hybrids between *An. gambiae* and *An. coluzzii* (ie, formerly referred to as M/S hybrids) was also shown to vary both geographically and temporally throughout their common distribution area in West and Central Africa [10,11,25,26]. The ecological determinants of such a dynamic hybridization process are currently unknown and may be under the control of a few genes mapping in areas of high genomic divergence between *An. coluzzii* and *An. gambiae* that have been called ‘speciation islands’ [2,3,22]. This is the case of Or39 lying in a genomic region of high differentiation on chromosome 2R. Indeed, our results detected high levels of genetic differentiation throughout this gene among the sympatric mosquito populations we have sampled, with fixed differences in coding regions resulting in amino-acid changes in the encoded protein. There was however no sign of diversifying selection acting on this gene, at least in our limited dataset which resulted in low statistical power of neutrality tests. Meanwhile, the three species explored in this study exhibited different mature peptides. Most of the aminoacid changes observed between species were located on the intracellular domains of the protein, and might indeed reflect random changes accumulated since lineage splitting within the *An. gambiae s.l.* complex. However, the nucleotide sequence of the first, and longest (*i.e.,* 72 aminoacids) extracellular domain of the protein was different between species, and polymorphic within the *An. gambiae* and *An. arabiensis* samples. At this stage of our analysis, we can only speculate on the role these amino acid changes may play for the perception of different olfactory stimuli by the mosquitoes through specific ligand*-*receptor interactions, thereby enhancing reproductive isolation when disruptive selective forces apply. The molecular mechanisms that are involved in such processes will have to be investigated, as well as their biological relevance to be evaluated through functional studies. Moreover, further knowledge on the mechanisms involved in mating behavior and the genetics and ecology of mate choice in this major disease vector is needed for the development of alternative vector control strategies based on population replacement/suppression.

### Availability of supporting data

The data set supporting the results of this article is available in the treebase repository under reference 15820; http://purl.org/phylo/treebase/phylows/study/TB2:S15820.

## Competing interests

The authors declare that they have no competing interests.

## Authors’ contributions

PK, AD, KRD and FS initiated and designed the study. HB and PK organized and conducted the field work. PK, CB and AA carried out lab work and analyzed data. AA, PK and FS wrote the manuscript. All authors read, revised and approved the final manuscript.
